# Predictive ability of the health belief model in HIV testing and counselling uptake among youth aged 15–24 in La-Nkwantanang-Madina Municipality, Ghana

**DOI:** 10.1186/s12889-024-19362-4

**Published:** 2024-07-09

**Authors:** Rosemond Appau, Richard Gyan Aboagye, Morkporkpor Nyahe, Nelisiwe Khuzwayo, Elvis Enowbeyang Tarkang

**Affiliations:** 1 School of Public Health, University of Health and Allied Sciences, Ho, Volta Region Ghana; 2 HIV/AIDS Prevention Research Network Cameroon, Kumba Southwest Region, Cameroon; 3 School of Nursing and Public Health, University of KwaZulu-Natal, Durban, South Africa

**Keywords:** HIV testing and counselling uptake, Health Belief Model, Youth, Ghana

## Abstract

**Background:**

Majority of new Human Immunodeficiency Virus (HIV)-positive persons in Ghana are aged 15–24. HIV prevalence among persons aged 15–24 years, a proxy for new infections, remained stable at 1.5% for 2017 and 2018, making it a significant public health concern. Yet only 26.4% of females and 8.6% of males aged 15–24 years know their HIV status. This study determined the predictive ability of the Health Belief Model (HBM) in HIV testing and counselling (HTC) uptake among youth (15–24 years) in the La-Nkwantanang Madina Municipality, Ghana.

**Methods:**

A cross-sectional design was adopted for the study, using a multistage sampling method to select 415 youth aged 15–24. Data were collected using a structured interviewer-administered questionnaire, and analysed using binomial logistic regression with STATA software version 16.0 at *p* < 0.05 significance level and at 95% confidence interval.

**Results:**

HTC uptake was 29.2%. Perceived susceptibility, perceived barriers, and perceived self-efficacy predicted HTC uptake. Youths with a high-risk perception for contracting HIV [OR = 3.03; 95% CI = 1.46, 6.30, *p* = 0.003], who perceived that they can contract HIV if not protected [OR = 3.69; 95% CI = 1.47, 9.22, *p* = 0.005], and worry about getting HIV [OR = 3.03, 95% CI = 1.61, 5.69, *p* < 0.001] (perceived susceptibility) were more likely to uptake HTC. Youths who had no trust issues with health workers [OR = 3.53; 95% CI = 1.46, 8.53, *p* = 0.005] and those who were not afraid of positive HIV test results [OR = 5.29; 95% CI = 2.66, 10.51, p,0.001] (perceived barriers) were more likely to uptake HTC. Youths who had no difficulties in turning up for appointments (perceived self-efficacy) had higher odds of HTC uptake [OR = 11.89, 95% CI = 6.73, 20.98, *P* < 0.001]. For the modifying factors, being married [OR = 2.96; 95% CI = 1.65–5.33], and having knowledge of HTC [OR = 9.10; 95% CI = 2.16–38.3], significantly influenced HTC uptake.

**Conclusion:**

Health promotion interventions to increase HTC uptake should focus on heightening the perception of susceptibility to HIV, reducing the barriers to HTC uptake, and increasing the self-efficacy for HTC uptake. The interventions should also target the significant modifying factors.

**Supplementary Information:**

The online version contains supplementary material available at 10.1186/s12889-024-19362-4.

## Background

Human Immunodeficiency Virus (HIV) testing and counselling (HTC) remains an innovative preventive strategy and the starting point to the care, treatment, and rehabilitation of HIV/Acquired Immune Deficiency Syndrome (AIDS) patients [1]. Approximately 38.4 million people were living with HIV globally with 1.5 million newly infected cases in the year 2021 [2]. Also, an estimated 3.4 million youth (15–24 years) are living with HIV globally [3]. The African Region is the most severely affected with two-thirds (25.6 million) of all people living with HIV (PLHIV) globally as of 2022 [4]. In sub-Saharan Africa (SSA), over 65% of youth aged 15–24 years are not aware of their status, indicating that a substantial number of youths in this age group are undiagnosed for HIV despite their high-risk sexual behaviours [5, 6].

In Ghana, there were 342,307 PLHIV in 2019, of which 20,068 were newly infected [7]. Out of the newly infected individuals, 5,613 (28%) were youth aged 15–24 years. Data from the 2022 Ghana Demographic and Health Survey [GDHS] showed that 54% of women and 24% of men aged 15–49 years had ever tested for HIV and received their results. Out of this, the uptake of HTC among those aged 15–24 was 27.8% for females and 8.3% for males [8]. Also, data from the Ghana AIDS Commission (GAC) showed that more than half of new HIV-positive persons were aged 15–24 years [9]. Generally, most youth express little or no willingness to undertake HIV testing due to psychological and personal factors [10, 11].

Regional and Municipal disparities in HIV prevalence exist in Ghana with the greatest often found among those in populated surroundings and urban cities of which La-Nkwantanang Madina Municipality is not an exception. For instance, evidence suggests that the regions with the highest and lowest HIV prevalence were Ahafo (2.66%) and North East (0.39%) respectively [7]. At the municipal/district level, HIV prevalence ranged from 5.56% in the Lower Manya Krobo as the district with the highest to 0.07% in Karaga and Tolon as districts with the lowest prevalence [9]. La-Nkwantanang Madina Municipality contributed 6.6% of the national HIV prevalence [7]. However, evidence on the proportion of youth who had tested for HIV remains sparse.

To end the HIV epidemic, the 90-90-90 target was established by the United Nations (UN), meaning that by the end of 2020, 90% of PLHIV should be aware of their status, 90% of HIV patients diagnosed should have access to antiretroviral therapy (ART), and 90% of those receiving ART should have viral suppression [1, 12]. Statistics from the GAC show that in 2020, Ghana achieved 63.2% in the first 90, 60.3% in the second 90 and 44.0% in the third 90, with key challenges such as stigma, discrimination, abandoning of treatment for prayer camps and false claims of cure [13].

Attempts to reach this overarching goal and eventually the Sustainable Development Goal 3.3 (end the epidemic of AIDS, tuberculosis, malaria, and neglected tropical diseases and combat hepatitis, water-borne disease, and other communicable diseases) by the end of 2030 [14], persists as a major concern given prevailing institutional and programmatic challenges [1, 7]. Ghana has subscribed to the UNs’ agenda of ending the epidemic of HIV/AIDS by 2030 backed by the National HIV and AIDS Policy [7]. HTC is freely available in all health facilities as a measure to ensure that everyone gets tested to know their status. However, HTC uptake remains low in Ghana [15]. For instance, 38.0% of young people 15–39 years in Kumasi [16], 65.7% of tertiary students in Ho Municipality [17] and 30.6% of tertiary students in Hohoe Municipality [18] had ever tested for HIV. Low uptake of HTC has implications for HIV transmission and treatment. For instance, knowing one’s serostatus through HTC can encourage safer sexual conduct. As one important pillar of HIV treatment still remains HTC uptake, those who test positive for HIV may be eligible for antiretroviral medication to reduce transmission rate and mortality [16].

Factors associated with low HTC uptake include issues of confidentiality of results [11, 18], stigma and discrimination [10, 19], fear of positive results [20], and distance to HTC centres [20, 21]. Also, an individual’s knowledge of HIV transmission and availability of HTC services [16, 21, 22] coupled with age [18, 22], sex [16], place of residence [23], marital status [16, 23], and education [24] are major factors. Previous studies have indicated that individuals who fear that their status might be made known to others after testing for HIV were less likely to undertake HTC [11, 25]. Other individuals also consider the distance to the health facility as a factor whereby those closer to health facilities had a higher likelihood of testing relative to those distant from the facilities [21]. An individual’s level of education and knowledge of HIV and availability of HTC services increased their likelihood of HTC uptake [21, 22, 24].

### Theoretical framework

The current study was grounded on the Health Belief Model. The HBM is a psychological model that was developed in the 1950s by social psychologists working in the Public Health Service, United States to better understand the widespread failure of a free Tuberculosis (TB) health screening programme [26, 27]. HBM has been used widely since then in health behaviour research to explain and predict health behaviour [26]. The areas the model has been adapted to include long- and short-term health behaviours such as sexual risk behaviours and HIV/AIDS transmission, and breast screening programmes [26].

The model has six (6) constructs that aid in explaining and predicting health behaviours. The constructs include perceived susceptibility, perceived severity, perceived benefits, perceived barriers, cues to action, and perceived self-efficacy. These constructs can be grouped into three categories: individual perceptions, modifying factors, and likelihood of action. Individual perceptions are factors that affect the perception of illness or disease, they deal with the importance of health to the individual; They include perceived susceptibility and perceived severity. Modifying factors include demographic and structural variables. The likelihood of action discusses factors in the probability of appropriate health behaviour; it is the likelihood of taking the recommended preventive health action [27].

In terms of its application to HTC, the model proposes that a person’s likelihood of engaging in particular health behaviour (HTC) is influenced by his or her perceived susceptibility to the health outcome (the subjective perception of the risk of contracting HIV), the perceived seriousness of the health outcome (one’s feelings about the clinical, medical and social consequences of living with HIV/AIDS), the perceived benefits of preventive actions (being tested for HIV), the perceived barriers or costs of taking a certain health action (tangible and psychological costs associated with getting HIV test), cues to action or cues that prompt one to take a certain action (information and media campaigns encouraging HIV testing); and self-efficacy or perceived confidence in taking a particular health action to mitigate the health condition [28].

### Individual perception

According to Tarkang and Zotor [29], individual perceptions are a person’s beliefs about one’s susceptibility to a disease together with the seriousness with which one views the perceived threat to the disease. In this study, the individual perceptions concern youth beliefs about their susceptibility to HIV/AIDS and their perceived severity of HIV/AIDS. However, their perception can be changed through knowledge acquisition. Perceived susceptibility refers to a person’s belief about the chances of contracting a health condition [29]. In the present study, perceived susceptibility refers to a youth’s (15–24 years) evaluation of their chances of getting HIV/AIDS. On the other hand, perceived severity can be defined as an individual’s belief about how serious a condition and its consequences are [29]. It refers to a youth evaluation of how serious HIV/AIDS is, its treatment, what its consequences would be (individual, societal, family), and whether the consequences are significant enough to avoid [30].

### Likelihood of action

Perceived benefit is an individual’s belief in the efficacy of the advised action to reduce risk or seriousness of impact [29]. For this study, refers to a youth evaluation of how well an advised or recommended action will reduce their risk of contracting HIV or moderate the impact of HIV/AIDS such as knowing their status through HIV testing [30].

Perceived barriers refer to an individual’s belief in the tangible and psychological cost of the advised behaviour [29]. In this study, perceived barriers entail an individual’s evaluation of the challenges or hindrances that might prevent them from undergoing HTC, which can be monetarily, emotional, psychological, or social [30].

Cues to action refer to the evidence or experiences either personal, interpersonal, or environmental that motivate a person to initiate an action [29]. In the present study, cues to action will be defined as events strategies or awareness initiatives that increase motivation or influence youth to undertake HIV testing.

Perceived self-efficacy is an individual’s confidence in their ability to successfully act [29]. It refers to a youth’s confidence in their ability to undertake HIV testing to know their status in this study.

### Modifying factors

**Fig. 1:**
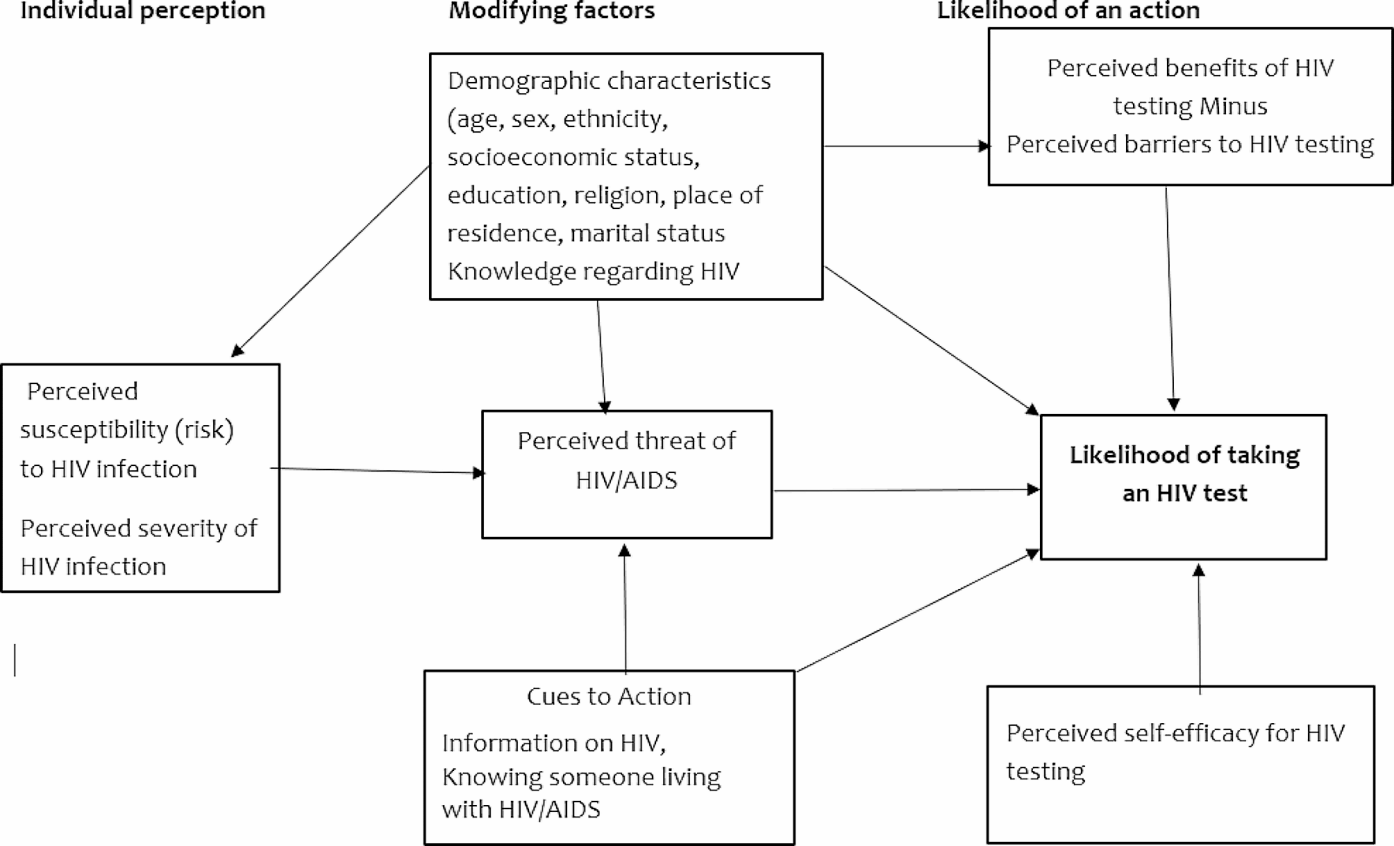
Health belief model conceptual framework for HIV testing and counselling uptake [27]

Modifying factors are the variables or characteristics that can influence an individual’s perceptions and indirectly influence health-related behaviours [29]. These factors (age, gender, marital status, geographical location, education, knowledge regarding HIV/AIDS) can affect a person’s perception of susceptibility to contracting HIV, severity of suffering from HIV/AIDS and its complications, and one’s perceived benefits to be expected from knowing their HIV status [29]. Figure 1 shows the conceptual framework of the study.

## Methods

### Study site

The study was conducted in the La-Nkwantanang Madina Municipality. The Municipality is located in the Northeastern part of the Greater Accra Region [31]. The Municipality has an estimated population of 130,380 with an annual growth rate of 4.2%. The majority (82.0%) of the inhabitants of the Municipality live in urban/peri-urban towns [32]. There are several health facilities in the Municipality consisting of 19 Community-Based Health Planning and Services (CHPS), 2 polyclinics, a health center, a Psychiatric Hospital, 4 public facilities, and 19 private facilities. The Pentecost Hospital, a Christian Association of Ghana health facility is the main Hospital serving the Municipality [32]. All the health facilities in the Municipality provide HTC services. La-Nkwantanang Madina Municipality was chosen as the study site due to its high contribution to the national HIV prevalence [7] coupled with the lack of data on HTC uptake among youth aged 15–24.

### Study design

A descriptive community-based cross-sectional design was adopted for this study. This design allows for easy accessibility to the respondents. Also, the cross-sectional design allows for the collection of data on outcome and their exposures simultaneously.

### Study population

The study consisted of youth aged 15–24 years in the La-Nkwantanang Madina Municipality.

### Inclusion and exclusion criteria

The study included youth aged 15–24 years who were residing in the Municipality for not less than six months. Youth who were either sick or mentally challenged or had not consented to participate in the study were excluded from the study.

### Sample size determination

The sample size for cross-sectional studies was calculated using the formula: $\:n\:=\frac{{z}^{2}\times\:p\left(q\right)}{{d}^{2}}\:$ [33]. Where, n = required sample size, z = reliability coefficient (z-score) of 1.96 at a 95% confidence level, q = 1-p, p = prevalence of HTC uptake from a previous study, and d = margin of error of 5% (0.05). With a 0.455 (45.5%) prevalence of HTC uptake from a previous study among youth aged 15–24 in Techiman, Ghana [15], the sample size was calculated as follows:

$$\begin{gathered}\:{\text{n}} = \:\frac{{{{1.96}^2}{\text{x}}\:0.455\:(1 - 0.455)}}{{{{0.05}^2}}} \hfill \\= \:\frac{{{{1.96}^{2\:\:\:\:}}{\text{x}}\:0.455\:\left( {0.545} \right)}}{{{{0.05}^2}}},\,{\text{n}} = 381 \hfill \\ \end{gathered}$$

Adjusting for a 5% non-response rate, $\:\:{\text{n}} = \left( {\frac{5}{{100}}{\text{x}}\:381} \right) = 19.05$

The minimum sample size for the study was 381 + 19.05 = 400 youth. However, 415 youth were recruited for the study.

### Sampling method

A multistage sampling technique was employed in the recruitment of respondents. All three sub-Municipalities were selected first where each sub-Municipality was treated as a stratum. Using the total population of the Municipality and that of the sub-Municipalities, the estimated sample size for each stratum was calculated as the total population for a stratum divided by the total population for the Municipality, all multiplied by the sample size for the study. The result was then recorded as the sample size for a particular stratum and this process continued till all the sample sizes for the remaining strata were obtained. The sample sizes obtained for each of the strata were Madina (170), Damfa (111), and Pantang (134). In each of the selected strata, a simple random sampling method conducted through balloting without replacement was used to select three communities from the list of all communities. Thus, a total of nine communities were selected for the study.

At the community level, a systematic sampling technique was used to select the households serially. A list of households obtained from the La-Nkwantanang Madina Municipal Assembly served as the sampling frame for each selected community. Systematic random sampling was used to select households from each community. This involved estimating the sampling interval (K^th^) as N/n, where N is the number of households in the community and n is the required sample size for the community. Pieces of paper of equal sizes were numbered from 1 to K^th^, placed in a box, and thoroughly mixed.

A piece of paper was randomly selected from the box to determine the starting point on the list for selecting the households for the study. The randomly selected number (N^th^) between 1 and K^th^ was the start point from which every K^th^ household on the list was selected. The calculated K^th^ for the current study was 5; therefore, every fifth household from N^th^ was selected. The fourth stage was the selection of the participants for the study. In each household, any youth who met the inclusion criteria and consented to participate was included. In households with more than one eligible youth, a simple random sampling technique was used to select one respondent. Each household visited in each community was numbered until the sample size for that sub-Municipality was obtained. The same process was repeated in all the sub-Municipalities till the sample size for the study was obtained.

### Data collection procedure and instrumentation

The structured interviewer-administered questionnaire used in our study was developed from the review of literature that examine uptake of HTC [15, 17, 18, 28, 34]. Hence, pertinent variables from the literature were used to develop the questionnaire. The questionnaire was structured into five sections: sociodemographic characteristics, knowledge of HIV/AIDS, knowledge of HTC, utilisation of HTC services, and factors associated with HIV testing based on the constructs of the HBM. Three university graduates were employed and trained as data collectors. The data collectors were trained on consenting process, administering the questionnaire, and ensuring completeness of the questionnaire. The data collectors pretested the questionnaire in a different municipality not used for the study after which modifications were made for it to be used for this study. Written informed consent was obtained from youth aged 18 years and above. For those below 18 years, written child assent and parental or guardian consent were obtained before they were interviewed. Face-to-face interviews were used to administer the questionnaires to all the respondents. Data collection was done in April 2021.

### Measures

#### Dependent variable

HTC uptake was measured by asking the respondents if they had ever tested for HIV, with “Yes” and “No” responses. The percentage of “Yes” reported after the data analysis measured the prevalence of HTC uptake.

### Independent variables

The independent variables were the sociodemographic variables and the constructs of the HBM. Regarding the constructs of the HBM, all the items were measured on a 4-point Likert scale (Strongly agree, agree, disagree, and strongly disagree). During the statistical analysis a composite score was generated for each construct of the HBM: ‘strongly agree’ and ‘agree’ were considered as an agreement to an item while ‘strongly disagree’ and ‘disagree’ were considered as a disagreement to an item. The questionnaire used for the current study was uploaded as a supplementary file (Rosemond Questionnaire).

Cronbach’s Alpha was used to determine the reliability of the data collection instrument. As reported by Taber [35], Cronbach’s Alpha is used to the internal consistency of a group of questions on a questionnaire, particularly within group questions. The reliability of the items under each construct of the HBM was tested to ensure that they could be used to measure the construct adequately. The reliability of the entire HBM was also tested using Cronbach’s Alpha. A reliability coefficient of 0.70 and above is acceptable [35]. However, having a high value is not an indication that the items measured the same thing but rather there was a correlation with how the respondents answered the questions [35]. Evidence for construct validity was provided through the pretest and reliability tests.

The results show that the overall alpha coefficient for the entire HBM was 0.8042, ranging from 0.7246 (for perceived severity) to 0.8445 (for self-efficacy), which is adequate.

### Data analyses

Data were analysed using the STATA version 16 software program. Frequencies and percentages were used to present the results of the categorical variables. Binomial logistic regression was performed to determine the factors influencing HTC uptake. The results of the regression analysis were presented in tabular form using the log-likelihood ratios, chi-square, and pseudo-R-square. A p-value less than 0.05 was considered statistically significant. A Cronbach’s alpha test was also carried out to determine the reliability of each of the constructs of the HBM. Further, odds ratios (ORs) with their respective 95% confidence intervals (CIs) were reported to estimate the specific socio-demographic factors and HBM constructs predicting HTC uptake.

Multiple assumptions are to be met for a binomial logistic regression to be performed. Some of those assumptions are that the dependent variable is dichotomous (uptake of HTC: ‘yes/no’) and that no outliers are to be present. Other assumptions include one or more independent variables that are measured on a categorical or continuous scale and there should be 15 cases per independent variable [36]. These assumptions were met in the current study, thereby justifying the use of binomial logistic regression.

In this paper, the HBM is tested drawing on its relevant assumptions, with regard to HTC uptake. The aim was to retain the assumptions of the model as much as possible and to assess the contributions of each component of the HBM and the various combinations of the components concerning HTC uptake. The different modelling alternatives considered were:

- Maintaining the assumptions of each component of the HBM.
- Full integration of all the components of the HBM.
- Integration of the components with high explanatory power.

Model estimation focused on mapping out the significant predictors of HTC uptake based on the significant constructs of the HBM. The dependent variable (HTC uptake) remained the same for all the modelling alternatives (the various constructs of the HBM).

## Results

### Sociodemographic characteristics of the respondents

The majority (57.1%, *n* = 237) were aged 15–19 years; most (52.5%, *n* = 218) were females; the majority (87.2%, *n* = 362) had never married; 192 (46.3%) had completed Senior High School (SHS); 148 (35.7%) belonged to the Akan ethnic group; 358 (86.3%) were Christians; 134 (32.3%) were living at Pantang; 165 (39.8%) were living in urban areas and most (72.5%, 301) were unemployed (Table 1).

### Knowledge of respondents of HIV/AIDS transmission

From Table 1, the majority (99.5%, *n* = 413) had heard about HIV/AIDS; most (98.3%, *n* = 408) agreed that a healthy person can be HIV-positive; that sexual activity with one uninfected partner reduces one’s risk of getting HIV (91.8%, *n* = 381); that HIV cannot be transmitted by mosquito bite (96.9%, *n* = 402); that HIV cannot be transmitted through supernatural means (96.6%, *n* = 401), and that HIV cannot be contracted through sharing food with an infected person (96.9%, *n* = 402). The majority (63.4%, *n* = 264) agreed that consistent condom use reduces one’s chances of acquiring HIV. The majority (92.0%, *n* = 382) had an overall adequate knowledge of HIV/AIDS transmission, while most (90.1%, *n* = 374) knew about HTC.

**Table 1:** Sociodemographic characteristics and knowledge o*f* HIV/AIDS transmission of the respondents (*N* = 415)

Sociodemographic characteristics		
Variable	Frequency (*n*)	Percentage (%)
**Age (group)**		
15–19	237	57.1
20–24	178	42.9
**Sex**		
Male	197	47.5
Female	218	52.5
**Marital status**		
Never married	362	87.2
Married	53	12.8
**Level of education**		
No education	14	3.4
Primary	18	4.3
JHS	107	25.8
SHS	192	46.3
Tertiary	84	20.2
**Ethnicity**		
Akan	148	35.7
Ewe	109	26.3
Ga/Dangbe	84	20.2
Northerners	74	17.8
**Religious affiliation**		
Christianity	358	86.3
Islamic	57	13.7
**Location of residence**		
Damfa	111	26.8
Nkwantanang	67	16.1
Pantang	134	32.3
Rawlings Circle	39	9.4
Social Welfare	64	15.4
**Type of settlement**		
Urban	165	39.8
Semi-rural	155	37.3
Rural	95	22.9
**Employment status**		
Unemployed	301	72.5
Self-employed	62	14.9
Privately employed	33	8.0
Government employee	19	4.6
**Knowledge of** ** HIV/AIDS and its transmission**		
**Heard about HIV/AIDS**		
No	2	0.5
Yes	413	99.5
**A healthy person can have the HIV/AIDS virus**		
No	7	1.7
Yes	408	98.3
**Consistent condom use reduces chances of HIV infection**		
No	151	36.4
Yes	264	63.6
**Having one faithful uninfected sexual partner reduces one’s chances of HIV infection**		
No	34	8.2
Yes	381	91.8
**HIV can be transmitted by the bite of a mosquito**		
No	402	96.9
Yes	13	3.1
**HIV can be transmitted by supernatural means**		
No	401	96.6
Yes	14	3.4
**HIV can be transmitted through sharing food with an infected person**		
No	402	96.9
Yes	13	3.1
**Overall knowledge of HIV/AIDS transmission**		
No	33	8.0
Yes	382	92.0
**Overall knowledge about HTC**		
No	41	9.9
Yes	374	90.1

JHS = Junior High School; SHS = Senior High School

### Uptake of HIV voluntary testing and counselling and constructs of the Health Belief Model

The uptake of HTC among the respondents was 29.2%. Table 2 presents the results of the constructs of the HBM. All the constructs of HBM were assessed using multiple questions. Regarding perceived susceptibility, the majority (67.5%, *n* = 280) perceived that they are at high risk of contracting HIV; most (89.4%, *n* = 371) agreed that it is possible for them to contract HIV at some point in time if they do not protect themselves and 250 (60.2%) were worried about contracting HIV. Regarding perceived severity, most (93.3%, *n* = 387) perceived HIV as a serious condition that one has to live with for life; the majority (95.9%, *n* = 398) believed HIV is a severe health problem, and most (96.4%, *n* = 400) believed that HIV will interfere with their social life. Regarding perceived benefits, the majority (97.1%, *n* = 403) believed that it is important to know their HIV status; most (94.5%, *n* = 392) believed that it is easy for an HIV patient to get AIDS medication and 399 (96.1%) believed that they will get adequate treatment if diagnosed with HIV. Regarding perceived barriers, the majority (91.6%, *n* = 380) perceived that they will be stigmatised and discriminated against if family and friends know that they are HIV-positive; 284 (68.4%) believed health workers cannot be trusted as they can leak out their test results; 360 (86.7%) believed that the nearest testing centre is far away from where they reside; 240 (57.8%) were afraid of an HIV-positive test result and 356 (85.8%) perceived that the location of the testing centre is not convenient for them. Regarding cues to action, only 19 (4.6%) mentioned that knowing someone who has tested for HIV motivated them to do the test; 17 (4.1%) mentioned that hearing about HTC from friends and family members motivated them to do the test; 20 (4.8%) mentioned that knowing someone who died from AIDS motivated them to do the test and 401 (96.6%) mentioned that they always hear about HTC from the mass media. Regarding perceived self-efficacy, the majority (97.6%, *n* = 405) believed that they are confident to utilise HTC services; 406 (97.8%) believed they can arrange for an HIV test if they wanted to and 205 (49.4%) believed that they would find it difficult to turn up for an appointment to do an HIV test.

**Table 2:** Constructs of the health belief model

Variables	Frequency (*n*)	Percentage (%)
**Perceived susceptibility**		
I am at high risk of contracting HIV		
Disagree	135	32.5
Agree	280	67.5
It is possible that I can contract HIV at some point in time if I do not protect myself		
Disagree	44	10.6
Agree	371	89.4
I worry a lot about getting HIV		
Disagree	165	39.8
Agree	250	60.2
**Perceived severity**		
I believe HIV is a severe health problem		
Disagree	17	4.1
Agree	398	95.9
HIV is a serious condition that I will live with for life		
Disagree	28	6.7
Agree	387	93.3
HIV will interfere with my social life		
Disagree	15	3.6
Agree	400	96.4
**Perceived benefits**		
It is important to know my HIV status so that if I am positive, I will not infect others		
Disagree	12	2.9
Agree	403	97.1
It is easy for people with HIV to get AIDS medication		
Disagree	23	5.5
Agree	392	94.5
I will receive adequate treatment if I am tested positive for HIV		
Disagree	16	3.9
Agree	399	96.1
**Perceived barriers**		
I will be stigmatised or discriminated against if family or friends get to know that am HIV-positive		
Disagree	35	8.4
Agree	380	91.6
Health workers cannot be trusted as they can leak out my test result		
Disagree	131	31.6
Agree	284	68.4
The HIV testing centre is far away from my place of residence		
Disagree	55	13.3
Agree	360	86.7
I am afraid of HIV-positive test result		
Disagree	175	42.2
Agree	240	57.8
The location of the HIV testing centre is not convenient for me		
Disagree	59	14.2
Agree	356	85.8
**Cues to action**		
Knowing someone who had tested for HIV motivated me to undertake an HIV test		
Disagree	396	95.4
Agree	19	4.6
Hearing about HIV voluntary testing and counselling from friends and family made me undertake an HIV test		
Disagree	398	95.9
Agree	17	4.1
Knowing someone who died of HIV made me undertake a test to know my status		
Disagree	395	95.2
Agree	20	4.8
I often hear about HIV testing to know my status on mass media		
Disagree	14	3.4
Agree	401	96.6
**Perceived self-efficacy**		
I am confident that I can use HIV testing services		
Disagree	10	2.4
Agree	405	97.6
I could arrange to have an HIV test if I want to		
Disagree	9	2.2
Agree	406	97.8
If I want an HIV test, I will find it difficult to turn up for the appointment		
Disagree	210	50.6
Agree	205	49.4

### Predictive ability of the health belief model in HIV testing and counselling uptake

Table 3 exhibits the results of the association between the components of the HBM and HTC uptake. Most of the constructs including the Integrated Value Mapping (IVM) had a Cronbach’s alpha above 0.7.

Table 3 further revealed that the highest explanatory power of the model was recorded in the IVM (combination of all models) (37.1%) followed by IVM (excluding the 3 insignificant constructs [perceived severity, perceived benefits, and cues to action] and the modifying factors) (22.9%). Among the constructs of the HBM, the highest explanatory power was found among perceived self-efficacy (20.3%), and modifying factors (19.2%), followed by perceived barriers (17.1%) and perceived susceptibility (12.4%). The level of significance of the constructs of the HBM also followed the same pattern as their explanatory powers. Perceived susceptibility (*p* < 0.001), perceived barriers (*p* < 0.001), perceived self-efficacy (*p* < 0.001), modifying factors (*p* < 0.001), and the two IVMs (*p* < 0.001) were the significant predictors HTC uptake.

**Table 3:** Association between the constructs of the HBM and uptake of HIV testing and counselling

Model component	LR Chi-square	df	*P*-value	Pseudo *R*-Square	Explanatory power of the model	Reliability analysis (Alpha)
1. Perceived susceptibility to HIV/AIDS	61.91	3	< 0.001*	0.1236	12.4%	0.7756
2. Perceived severity to HIV/AIDS	2.35	3	0.503	0.0047	0.5%	0.7246
3. Perceived benefit of HIV testing and counselling	7.36	3	0.061	0.0147	1.5%	0.7710
4. Perceived barriers to HIV testing and counselling	85.60	5	< 0.001*	0.1709	17.1%	0.7587
5. Cues to action	5.96	4	0.202	0.0119	1.2%	0.8140
6. Perceived self-efficacy	101.80	3	< 0.001*	0.2032	20.3%	0.8445
7. Modifying factors	96.14	22	< 0.001*	0.1919	19.2%	0.3084
8. Integrated value mapping (IVM) (combination of all models)	186.02	43	< 0.001*	0.3713	37.1%	0.8042
9. Integrated value mapping (IVM) (excluded components 2, 3, 5, 7)	114.67	11	< 0.001*	0.2289	22.9%	0.8334

*= *p* < 0.001; df = Degree of freedom

### Likelihood ratio test of the items under the constructs of the HBM regarding HTC uptake

Table 4 presents the results of the binomial logistic regression for the association between the constructs of the HBM with satisfactory explanatory power and HTC uptake. For perceived susceptibility, youths who had a high-risk perception for contracting HIV [OR = 3.03; 95% CI = 1.46, 6.30, *p* = 0.003], who perceived that they can contract HIV if not protected [OR = 3.69; 95% CI = 1.47, 9.22, *p* = 0.005], and those who worry about getting HIV [OR = 3.03, 95% CI = 1.61, 5.69, *p* < 0.001] were more likely to uptake HTC. Regarding perceived barriers, youths who had no trust issues with health workers [OR = 3.53; 95% CI = 1.46, 8.53, *p* = 0.005] and those who were not afraid of positive HIV test results [OR = 5.29; 95% CI = 2.66, 10.51, p,0.001] were more likely to undergo HTC. Concerning perceived self-efficacy, youths who had no difficulties in turning up for appointments had higher odds of HTC uptake compared to those who had [OR = 11.89, 95% CI = 6.73, 20.98, *P* < 0.001].

**Table 4:** Likelihood ratio test of the items under each construct of the HBM regarding HIV testing and counselling uptake

Variables	-2 Log Likelihood of Reduced Model	Chi-square	df	*p*-value	OR [95% CI]
**Perceived susceptibility**					
I am at high risk of contracting HIV	229.479	41.99	1	< 0.001	3.03 [1.46, 6.30] 0.003
It is possible that I can contract HIV at some point in time if I do not protect myself	247.204	6.54	1	0.011	3.69 [1.47, 9.22] 0.005
I worry a lot about getting HIV	229.290	41.76	1	< 0.001	3.03 [1.61, 5.69] < 0.001
**Perceived barriers**					
I will be stigmatised or discriminated against if family or friends get to know that am HIV positive	248.152	4.64	1	0.031	0.57 [0.17, 1.85] 0.345
Health workers cannot be trusted as they can disclose my test results	223.253	54.44	1	< 0.001	3.53 [1.46, 8.53] 0.005
The HIV testing centre is far away from my place of residence	247.706	5.53	1	0.019	1.81 [0.75, 4.34] 0.187
I am afraid of HIV positive test results	215.907	69.13	1	< 0.001	5.29 [2.66, 10.51] < 0.001
The location of the HIV testing centre is not convenient for me	250.235	0.47	1	0.491	1.64 [0.79, 3.37] 0.183
**Perceived self-efficacy**					
I am confident I can use HIV testing services	249.367	2.21	1	0.137	3.61 [0.35, 37.70] 0.284
I could arrange to have an HIV test if I want to	249.606	1.73	1	0.188	2.05 [0.17, 24.16] 0.569
If I want an HIV test, I will find it difficult to turn up for the appointment	201	98.74	1	< 0.001	11.89 [6.73, 20.98] < 0.001

OR = Odds ratio; CI = Confidence interval; df = Degree of freedom

### Association between modifying factors and HIV testing and counselling uptake

Table 5 presents the results of the association between the modifying factors and HTC uptake. Married youth were more likely to undergo HTC compared to the never-married counterparts [OR = 2.96; 95% CI = 1.65–5.33; *p* < 0.001]. The odds of HTC were higher among Ga/Dangbe youth compared to the Akans [OR = 3.14; 95% CI = 1.76–5.61; *p* < 0.001]. Muslims were 65% less likely to go for HTC than Christians [OR = 0.35; 95% CI = 0.16–0.77; *p* = 0.009]. Rural youth were more likely to undergo HTC compared to urban youth [OR = 5.22; 95% CI = 2.96–9.18; *p* < 0 001]. Youth who knew about HTC were more likely to undergo HTC [OR = 9.10; 95% CI = 2.16–38.3; *p* = 0.003].

**Table 5:** Modifying factors associated with uptake of HIV testing and counselling

Variable	-2 Log Likelihood of Reduced Model	OR (CI) *P*-value	Chi-square	df	*p*-value
***Modifying factors***					
***Age group***	250.444		0.06	1	0.810
***Sex***	250.415		0.11	1	0.736
***Marital status***	244.048		12.85	1	< 0.001
Married		**2.96 (1.65–5.33) < 0.001**			
***Level of education***	237.657		25.63	4	< 0.001
***Ethnicity***	242.312		16.32	3	< 0.001
Ga/Dangbe		**3.14 (1.76–5.61) < 0.001**			
***Religious affiliation***	246.347		8.25	1	0.004
Islam		**0.35 (0.16–0.77) 0.009**			
***Location of residence***	244.104		12.74	4	0.013
***Type of settlement***	232.332		36.28	2	< 0.001
Rural		**5.22 (2.96–9.18) < 0.001**			
***Employment status***	246.044		8.86	3	0.031
***Knowledge of HIV testing and counselling***	241.925		17.09	1	< 0.001
Yes		**9.10 (2.16–38.3) 0.003**			
***Knowledge on HIV/AIDS***	250.325		0.30	1	0.587

OR = Odds ratio; CI = Confidence interval; df = Degree of freedom

### Predictive ability of the Health Belief Model framework after analysis of the data

**Fig. 2:**
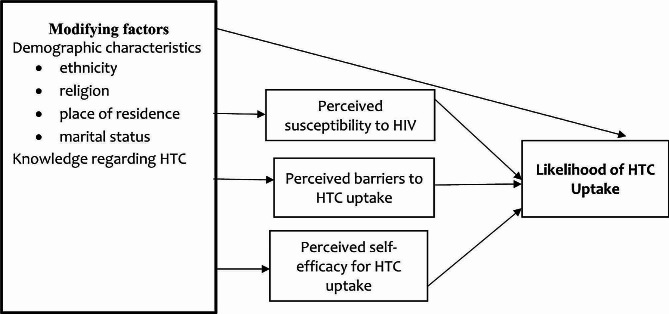
Predictive ability of the Health Belief Model regarding HIV Testing and Counselling uptake

From the current finding, the modifying factors (ethnicity, religion, place of residence, marital status & Knowledge regarding HTC), perceived susceptibility, perceived barriers and perceived self-efficacy predicted HTC uptake. Therefore, the assumptions of the HBM were partially supported by the current study. Figure 2 shows the framework of the constructs of the HBM that predict HTC from the current study.

## Discussion

The uptake of HTC was low (29.2%). HTC uptake in the present study is similar to the findings reported in previous studies conducted in Tarkwa-Nsuaem and Fanteakwa Districts, Ghana [37], the Hohoe Municipality, Ghana [18], Kenya [28] and Tanzania [20]. However, it is lower compared to findings from previous studies conducted in sub-Saharan Africa [22], Ethiopia [10], Ho Ghana [17], Accra Ghana [15], and Kumasi Ghana [16]. The plausible justification of the observed utilisation is that the facilities offering the HTC services may not be user-friendly, thereby leading to distrust between users and health workers. Also, deep-rooted societal perception of sexual promiscuity often associated with youth who utilise HTC services could hinder their ability to utilise these services [37]. The siting of the facilities coupled with some misconceptions about HTC could have also influenced the low HTC uptake.

Three constructs of the HBM: perceived susceptibility, perceived barrier and perceived self-efficacy significantly predicted HTC uptake. Regarding perceived susceptibility, all the items significantly predicted HTC uptake, which implies that the youth’s perception of their risk of contracting HIV is an important factor in HTC uptake. This finding is similar to those of studies conducted in China [38], Kenya [21] and Ethiopia [25, 34]. The youth in the current study might have utilised HTC services because of the perception of their risk of contracting HIV [34], or they might have perceived their risk of acquiring HIV to be higher relative to those with low risk perception [38]. The current finding is in line with the assumption of the HBM. According to the HBM [27], youth have to perceive that they are at risk of contracting HIV before they will be encouraged to utilise HTC services. Therefore, interventions to increase HTC uptake among the youths in the current study should focus on strategies to increase their perception of the risk of contracting HIV.

In line with the assumption of the HBM [27], items under perceived barriers (lack of trust in the health workers and fear of positive results) significantly predicted HTC uptake. The finding on fear of positive test results is consistent with the finding of a study conducted in South Africa [39]. Confidentiality, the siting of HTC center, and lack of trust among health workers have been reported in other studies as predictors of low HTC uptake [25, 39–41]. According to the HBM, youth in the current study should be equipped with strategies to overcome perceived barriers in order to utilize HTC services. This can be achieved by giving reassurances, providing incentives and correcting misperceptions regarding HIV and HTC. Given these barriers, Apanga et al. [24] highlighted the need for Ghana Health Service to revisit the channels used to deliver HTC services. There should be well-harmonised and integrated messages to alleviate misconceptions and instigate positive attitudes towards HTC uptake among the youth in La-Nkwantanang Madina Municipality.

In the current study, youth with limited self-efficacy (inability to turn up for HTC appointments) hindered their HTC uptake. Youth’s difficulty in turning up for appointments could be linked to barriers such as lack of trust and confidentiality, and fear of a positive result. Any youth who encounters any of these barriers during the initial visit might find it difficult to keep to the appointment for HTC uptake. Association between self-efficacy and HTC uptake has been reported in a study conducted in Malawi [19]. The current finding calls for interventions to provide youths with skills and training to enhance their self-efficacy for HTC uptake.

Concerning the modifying factors, HTC uptake was higher among those who were married, knew about HTC services, lived in rural areas, and belonged to the Ga/Dangbe ethnic group, but was lower among the Muslims. Those married were more likely to uptake HTC. This finding is consistent with those of previous studies conducted in Ghana [15], Ethiopia [42], Tanzania [43], Zambia [44], and Ethiopia [45]. Mahande et al. [43] highlighted that the higher HTC uptake among those married could be due to faith-based institutions advocating for the importance of having HIV testing before marriage and the compulsory counselling and testing promotion for couples who intend to marry in some religious organisations to help safeguard their marriage and the health of their future children [45].

Knowledge of HTC was associated with HTC uptake and this association has been reported in studies conducted in Kumasi Ghana [16], Ethiopia [10, 25], Tanzania [20], Kenya [21], and China [38]. This finding could be because knowledgeable individuals might perceive the benefits of HTC uptake. Additionally, the youth might have obtained educative and informative details on HTC from health workers or PLHIV, highlighting the essence of HTC uptake [25].

Muslims had lower odds of HTC uptake compared to Christians. This finding concurs with those of studies conducted in Ethiopia [46] and Ghana [47]. A possible explanation could be greater adherence to religious tenets, which may protect against HIV transmission through sexual contact. Even though polygamy is permitted for men and divorce is relatively more common in Islam, prohibitions against extramarital sex may outweigh the risks posed by unprotected sex [46]. A study conducted in Ghana showed that 21.55% of women have been in more than one union or marriage whilst 18.73% had co-wives in their marriage [48]. Hence, polygamous marriages are prevalent in the Ghanaian societies and more common among the Islamic communities.

Youth from rural areas were more likely to uptake HTC compared to those from urban areas. This finding is contrary to those of previous studies conducted in Ethiopia [40, 49] and Nigeria [50]. The current finding might have resulted from opportunistic testing and counselling through free screening carried out in those areas by benevolent individuals. Also, the youths might have been recommended to undergo HTC by health professionals upon assessment of their risky sexual behaviours or issues related to marriage or blood donation. Other studies have reported a significant association between youth risky sexual behaviour and a higher likelihood of HTC utilisation, which could explain the observed association in the current study [45, 46].

Youth who belonged to the Ga/Dangbe ethnic group were more likely to uptake HTC compared to Akan youth. Studies conducted in Mozambique [51] and Ethiopia [34] have reported ethnic variations in the likelihood of HTC uptake. Their finding suggests an influence of sociocultural differences that characterise different ethnic groups regarding HTC uptake. The belief that HIV/AIDS can be acquired through supernatural means and dependence on traditional healthcare-seeking among the Akan ethnic group could have influenced their uptake of HTC.

Perceived self-efficacy explained 20.3% of the variance in HTC uptake, followed by the modifying factors (19.2%), then perceived barriers (17.1%), and then perceived susceptibility (12.4%). These four constructs of the HBM were also the significant predictors of HTC uptake. The IVM including the significant perception constructs (perceived susceptibility, perceived barriers & perceived self-efficacy) put together, had an increased explanatory power of 22.9%.

Interventions to increase HTC uptake should focus on these three constructs together. The IVM of all the constructs of the HBM and the modifying factors put together gave an increased explanatory power of 37.1% and a significance of *p* < 0.001. Therefore, strategies to increase HTC uptake should also consider the significant modifying factors. These strategies should aim at increasing the perceived susceptibility to HIV, reducing the perceived barriers to HTC uptake, and increasing the self-efficacy for HTC uptake.

### Limitations of the study

Data were collected at the household, and community levels, therefore, there is the possibility that youths who were in school might have been missed. Additionally, the cross-sectional nature of the study was not appropriate to establish a causal relationship between outcome and explanatory variables. Also, other factors such as polygamy, history of risky sexual behaviour, and socioeconomic status could have influenced the results of the current study but were not assessed. Hence, the findings should be interpreted in line with the variables included in the study. Furthermore, sample weights were not used in the present study and this limit it’s generalisability to other youths in the La-Nkwantanang Madina Municipality.

## Conclusion

The study has shown that the perceived susceptibility, perceived barriers, and perceived self-efficacy significantly predicted HTC uptake among the youth in the La-Nkwantanang Madina Municipality. Health promotion interventions to increase HTC uptake should focus on heightening the perception of susceptibility to HIV, reducing the barriers to HTC uptake, and increasing the self-efficacy for HTC uptake. The interventions should also target the significant modifying factors identified in the study. Ghana Health Service should implement interventions such as targeted health promotion campaigns, awareness creation and education, community outreach programmes, and improvements in healthcare access to increase HTC uptake focusing on these significant factors.

### Electronic supplementary material

Below is the link to the electronic supplementary material.

Supplementary Material 1

## Data Availability

The datasets used and/or analysed during the current study are available from the corresponding author on reasonable request.
