# Supplementary material for: Predictive ability of the health belief model in HIV testing and counselling uptake among youth aged 15–24 in La-Nkwantanang-Madina Municipality, Ghana
Source: BMC Public Health. 2024 Jul 9;24:1825. doi: 10.1186/s12889-024-19362-4 (PMC11232218; doi:10.1186/s12889-024-19362-4)
Supplement: Supplementary file 1 — Supplementary Material 1 [file 12889_2024_19362_MOESM1_ESM.docx]

# APPENDIX I: QUESTIONNAIRE

Dear Respondent,

The purpose of the study is to determine the predictors of HIV Testing and Counselling uptake among youth aged 15-24 years in the La-Nkwantanang Madina Municipality, Ghana using the Health Belief Model. The information collected is solely for academic purpose. Confidentiality of responses will be ensured and names of the respondents will not be included in the final report.

**Participant code:** ………………... **Date:** …………………………….

***Please carefully read the questions or statements below and provide your responses by ticking/circling the appropriate response or by writing in the space provided***.

| **NO.** | **QUESTIONS/ITEMS** | **RESPONSE** |
| --- | --- | --- |
| **SECTION A: SOCIO-DEMOGRAPHIC CHARACTERISTICS** | | |
|  | What is your age in complete years? | …………………. |
|  | What is your date of birth? | …. /…. /…. (DD/MM/YY) |
|  | What is your sex? | 1. Male 2. Female |
|  | What is your location? | ……………… |
|  | What is your settlement type? | 1. Urban 2. Semi-rural 3. Rural |
|  | What is your marital status? | 1. single 2. Married 3. Divorced 4. Cohabiting 5. Widow |
|  | What is your ethnicity? | 1. Akan 2. Ewe 3. Ga/Dangbe 4. Hausa 5. Other (Specify) …………… |
|  | What is your religious affiliation? | 1. Christianity 2. Islam 3. African Traditional 4. Other (Specify) ……. |
|  | What is your highest level of education? | 1. None 2. Primary 3. Junior High school 4. Senior High school 5. Tertiary level |
|  | What is your employment status? | 1. Unemployed 2. Self-employed 3. Privately employed 4. Government employee |

| **NO.** | **QUESTIONS/ITEMS** | **RESPONSE** |
| --- | --- | --- |
| **SECTION B: KNOWLEDGE OF HIV/AIDS** | | |
|  | Have you heard about HIV/AIDS? | 1. Yes  2. No |
|  | Can a healthy person have the HIV/AIDS virus? | 1. Yes  2. No |
|  | Can consistent condom use reduce one’s chances of getting HIV? | 1. Yes  2. No |
|  | Can having sex with one uninfected faithful sexual partner reduces your one’s chances of getting the HIV? | 1. Yes  2. No |
|  | Can the HIV virus be transmitted by the bite of mosquito? | 1. Yes  2. No |
|  | Can the HIV virus be transmitted by supernatural means? | 1. Yes  2. No |
|  | Can a person become infected by sharing food with a person who has HIV/AIDS? | 1. Yes  2. No |
| **SECTION C: KNOWLEDGE OF HIV TESTING AND COUNSELLING** | | |
| 1. | Have you heard about HIV voluntary testing and counselling? | 1. Yes  2. No |
| 2. | If Yes, where did you hear about HIV testing and counselling? | 1. Health facility  2. Mass media  3. Family  4. Friends  5. Other (Specify)…… |
| 3. | Do you know where HIV testing and counselling services are provided? | 1. Yes  2. No |
| 4. | If yes, where is the HIV testing and counselling service provided? | 1. Government clinic/Hospital  2. Private clinic/Hospital  3. Voluntary testing centre  4. Other (specify)…… |
| **SECTION D: HIV TESTING AND COUNSELLING UTILIZAION** | | |
| 1. | Have you ever tested for HIV and know your status? | 1. Yes  2. No |
| 2. | When was the last time you had the test? | 1. Less than 3 months  2. 3- 6 months  3. 6months - 1 year  4. 1- 2 years  5. More than 2 years ago |
| 3. | If you had ever had HIV test, why did you undertake the test? | 1. To know my status  2. To get married  3. Blood donation requirement  4. Pregnancy or prenatal requirement  5. Concerns from my partner  6. Other (specify) ………… |
| 4. | If you have not had HIV test, why did you not undertake the test? | 1. Do not know where to go for the test  2. Not sexually active  3. Cost of test  4. Someone might see me  5. Other (specify) ………… |
| **SECTION E: INDIVIDUAL PERCEPTION INFLUENCING HIV TESTING BASED ON THE HEALTH BELIEF MODEL CONSTRUCTS** | | |
|  | **PERCEIVED SUSCEPTIBILITY** |  |
| 1. | I am at high risk of contracting HIV | 1. Strongly disagree 2. Agree 3. Disagree 4. Strongly disagree |
| 2. | It is possible that I can contract HIV at some point in time if I do not protect myself | 1. Strongly disagree 2. Agree 3. Disagree 4. Strongly disagree |
| 3. | I worry a lot about getting HIV | 1. Strongly disagree 2. Agree 3. Disagree 4. Strongly disagree |
|  | **PERCEIVED SEVERITY** |  |
| 1. | I believe HIV is a severe health problem | 1. Strongly disagree 2. Agree 3. Disagree 4. Strongly disagree |
| 2. | HIV is a serious condition that I will live with for life | 1. Strongly disagree 2. Agree 3. Disagree 4. Strongly disagree |
| 3. | HIV will interfere with my social life | 1. Strongly disagree 2. Agree 3. Disagree 4. Strongly disagree |
|  | **PERCEIVED BENEFITS** |  |
| 1. | It is important to know your HIV status, so that if you are positive you will not infect others | 1. Strongly disagree 2. Agree 3. Disagree 4. Strongly disagree |
| 2. | It is easy for people with HIV to get AIDS medication | 1. Strongly disagree 2. Agree 3. Disagree 4. Strongly disagree |
| 3. | I will receive adequate treatment if I am diagnosed positive | 1. Strongly disagree 2. Agree 3. Disagree 4. Strongly disagree |
|  | **PERCEIVED BARRIERS** |  |
|  | I will be stigmatised or discriminated against if family or friends get to know that am HIV-positive | 1. Strongly disagree 2. Agree 3. Disagree 4. Strongly disagree |
|  | Health workers cannot be trusted as they can leak out my results | 1. Strongly disagree 2. Agree 3. Disagree 4. Strongly disagree |
|  | The HIV testing centre is far away from my place of residence | 1. Strongly disagree 2. Agree 3. Disagree 4. Strongly disagree |
|  | I am afraid of HIV-positive test results | 1. Strongly disagree 2. Agree 3. Disagree 4. Strongly disagree |
|  | The location of the HIV testing centre is not convenient for me | 1. Strongly disagree 2. Agree 3. Disagree 4. Strongly disagree |
|  | **CUES TO ACTION** |  |
| 1. | Knowing someone who had tested for HIV motivated me to undertake an HIV test | 1. Strongly disagree 2. Agree 3. Disagree 4. Strongly disagree |
| 2. | Hearing about HIV voluntary testing and counselling from friends and family made me undertake an HIV test | 1. Strongly disagree 2. Agree 3. Disagree 4. Strongly disagree |
| 3. | Knowing someone died of HIV made me to undertake a test to know my status | 1. Strongly disagree 2. Agree 3. Disagree 4. Strongly disagree |
| 4. | I often hear about HIV testing to know my status on mass media | 1. Strongly disagree 2. Agree 3. Disagree 4. Strongly disagree |
|  | **PERCEIVED SELF-EFFICACY** |  |
|  | I am confident I can use HIV testing services | 1. Strongly disagree 2. Agree 3. Disagree 4. Strongly disagree |
|  | I could arrange to have an HIV test if I want to | 1. Strongly disagree 2. Agree 3. Disagree 4. Strongly disagree |
|  | If I want an HIV test, I will find it difficult to turn up for the appointment | 1. Strongly disagree 2. Agree 3. Disagree 4. Strongly disagree |

**Thank you very much for your time**
